# Correlation between paravertebral spread of injectate and clinical efficacy in lumbar transforaminal block

**DOI:** 10.1038/s41598-020-68474-5

**Published:** 2020-07-13

**Authors:** Yu Jeong Bang, Hue Jung Park, Woo Seog Sim, Dae Won Lee, Jin Young Lee

**Affiliations:** 1 Department of Anesthesiology and Pain Medicine, Samsung Medical Center, Sungkyunkwan University, School of Medicine, Seoul, 06351 Korea; 2 Department of Anesthesiology and Pain Medicine, Seoul St.Mary′s hospital, College of Medicine, The Catholic University of Korea, Seoul, 06591 Korea

**Keywords:** Outcomes research, Bone

## Abstract

The potential paravertebral space includes spinal nerves, dorsal rami, rami communicants, and sympathetic chains. This study evaluated correlations between paravertebral spread of injectate and clinical efficacy in lumbar transforaminal block. We retrospectively analysed the data of 88 patients who received transforaminal blocks for lumbar radicular pain. We categorized patients into two groups: patients with ≥ 50% pain reduction on a numeric rating scale at 30 min following a block (responder group), and patients with < 50% pain reduction (non-responder group). Paravertebral spread of injectate was graded as limited to the anterior, middle, and posterior 1/3 of the anterolateral aspect of vertebral bodies; spread between the posterolateral margins of bodies and the posterior epidural space was considered no spread. Clinical and fluoroscopic data, perfusion index, temperature, and cold sensation were compared between the groups. Among 54 patients analysed, 26 (48.1%) experienced ≥ 50% and 28 (51.9%) < 50% pain reduction. Paravertebral spread occurred in 33 (61.1%) patients; 19 (57.6%) responders and 14 (42.4%) non-responders. On analysis, paravertebral spread, epidural spread patterns, perfusion index change ratios, temperature changes, and cold sensation changes showed no differences between responder and non-responder groups. Paravertebral spread occurred in 61.1%, with no correlation with the clinical efficacy of lumbar transforaminal block.

## Introduction

The paravertebral (PV) space is a potential space on either side of the vertebral body, which contains spinal nerves, spinal dorsal rami, rami communicants, and sympathetic chains^1,2^. It is known to communicate with the epidural space through intervertebral foramina and with the contralateral PV space through the prevertebral or epidural spaces^1–3^. PV blocks are performed on all levels of the roots of nerves or plexuses to provide segmental anaesthetic and analgesic effects in the thorax, abdomen, pelvis, and lower limbs^4,5^. Although the PV space is defined as that within transverse process and intervertebral foramen, injectate spread to the PV space and its clinical efficacy has not yet been explored in cases of transforaminal block. In previous reports, epidural and spinal anaesthesia was presumed to attenuate sympathetic response due to PV spread; however, evidence is limited owing to the lack of methods for measuring sympathetic denervation^6–8^. Marhofer and colleagues reported that the intended effects of local anaesthetics were unpredictable and the effect was stronger than local anesthetic spread range in thoracic PV injection^2^. The variability of PV spread was suspected to be related to individual anatomic differences and/or secondary redistribution following injection^2^. The caudal boundary of the thoracic PV space is connected with the origin of the psoas major muscle^9^. Lumbar sympathetic ganglia are located on the anterolateral aspects of vertebral bodies L2, L2–3 disc, L3–4 disc, and L5^10,11^. Lumbar sympathetic block is indicated for the diagnosis and treatment of pain associated with sympathetic dysfunction, and is related to its prognosis^12,13^. The possibility of PV spread and subsequent sympathetic effects through transforaminal injection may be an important factor in deciding the indications for these injections in clinical practice. This study evaluated the correlation between the PV spread of injectate and clinical efficacy of lumbar transforaminal block.

## Methods

### Patients

We retrospectively reviewed the electronic medical records of 88 patients with lumbar radicular pain, who underwent transforaminal blocks between January and September 2019 at a single tertiary care hospital. The patients’ ages ranged from 31 to 85 years. All patients had lower back and radicular pain. The inclusion criteria were as follows: (a) a primary diagnosis of lower back pain radiating to the lower limbs and (b) a cross-sectional imaging study (either computed tomography [CT] or magnetic resonance imaging [MRI]) of the lumbosacral spine in patients diagnosed with spinal stenosis or herniated nucleus pulposus (HNP)^14^. The exclusion criteria included any history of lumbar surgery; lumbar neuroplasty; neoplastic diseases; peripheral vascular disease affecting pain, perfusion index and/or cold sensation; or use of medications affecting the vascular system^14^. We also excluded injections at L5, as the clinical response to the lumbar sympatholytic effect is known to be significant above the level of the L4 vertebral body^10,15^. The lesion level for transforaminal injections was selected on the basis of clinical manifestations, physical examination, and review of imaging studies^14^. Lesion severity was categorised as one of three different degrees (mild, moderate, or severe) by reviewing imaging data^14^.

### Interventions

**Figure 1:**
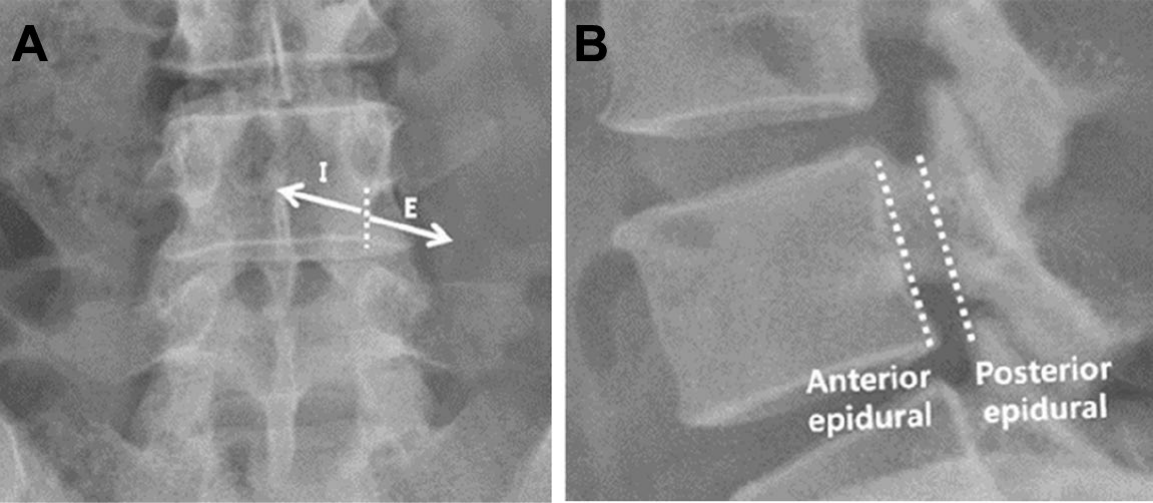
Schematic drawing for analysis of spread of injectate in the anteroposterior view. On anteroposterior view: (**A**) extra-foraminal (E), intra-foraminal (I), or extra and intra-foraminal (EI) spread based on 6 o’ clock location of the pedicle; on lateral view: (**B**) anterior or posterior epidural spread defined.

**Figure 2:**
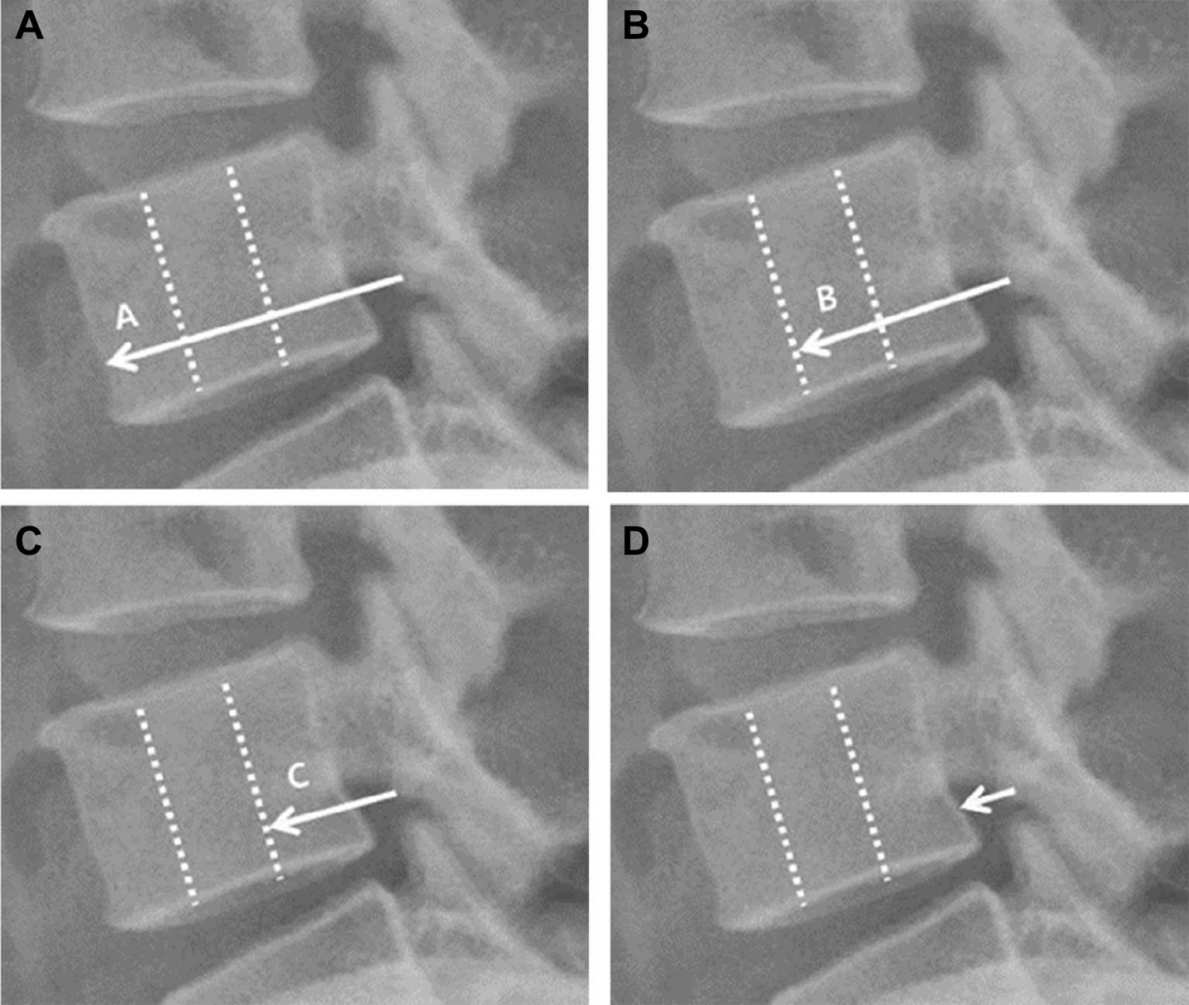
Schematic drawing for analysis of paravertebral spread of injectate. From posterior epidural space, (**A**) spread beyond anterior 1/3 of vertebral body, (**B**) spread to middle 1/3 of vertebral body, (**C**) spread to posterior 1/3 of vertebral body, (**D**) no spread (spread to posterior margin of the vertebral body).

All procedures were performed under fluoroscopic guidance in a standardised manner by a single experienced pain physician (J.Y.L). Patients were placed in the prone position, and anteroposterior (AP) and lateral view images were obtained using a C-arm (OEC series 9800, GE Healthcare, Chicago, Il) to ensure proper site of entry. Following aseptic preparation and application of 1% lidocaine, a 23-gauge Tuohy needle (Tae-Chang Industrial Co., Seoul, Korea) was passed through the skin overlying the upper quadrant of the target foramen. Aspirations were routinely performed to assess for the presence of blood or cerebrospinal fluid. On feeling a loss of resistance, the aspiration test was performed, followed by injection of 1–2 ml of contrast medium (Omnipaque®, 300 mgI.ml^-1^, GE Healthcare), which confirmed whether the point was well placed in the epidural space^14^. After confirming that the contrast had spread throughout the epidural space, a total volume of 3 ml (containing 1% lidocaine, dexamethasone, and contrast medium) was infused. The spread pattern of 3 ml of injectate was analysed based on the following criteria: posterior or anterior and posterior epidural spread on the lateral view, and extra-foraminal (E), intra-foraminal (I), or extra and intra-foraminal (EI) epidural spread on the AP view, based on a 6 o’clock location of the pedicle (Fig. 1). We assumed the presence of PV spread when the injectate was visible along the anterolateral aspect of the vertebral body; the spread was graded as that to the anterior 1/3 (A), middle 1/3 (B), and posterior 1/3 (C); when the injectate was shown behind the posterior margin of the vertebral body from the posterior epidural space on lateral view, it was considered as no spread (Fig. 2). Following the procedure, patients were observed for any adverse effects. The perfusion index (PI) was monitored using pulse oximetry (Root®, Mashimo Corporation, Irvine, CA) on the toe of the affected limb^14^. We assessed the PI prior to treatment (T0), 5 (T5), 15 (T15), and 30 (T30) min following transforaminal injection^14^. Temperature was assessed at T0, T5, T15, and T30 using a touch thermometer (IntelliVue MP70 patient monitor; Philips Healthcare, Best, the Netherlands) on the dorsum of the foot of the affected limb^14^. Room temperature was maintained at 23–25 °C. Pain was scored using a numeric rate scale (NRS), which ranged from 0 = no pain to 10 = absolutely intolerable pain; cold sensation of the affected limb (NRS: ranging from 0 = no cold sensation to 10 = most severe cold) was recorded at T0 and T30. The pain severity, PI, temperature, and cold sensation at T0 were recorded after 5 min of bed rest and before infiltrating the skin with 1% lidocaine^14^.

### Statistical analysis

All data were analysed using the SAS 9.4 (SAS Institute, Cary, NC) software package. The data have been presented as the mean ± standard deviation or number (proportion), as appropriate. Demographic data for the two groups were compared using the Chi-square, t-test, or Fisher’s exact tests. The injection level, injection side, epidural spread, and PV spread were compared using Fisher’s exact and Chi-square tests. For PV spread analysis, we compared spread with no spread, and graded spread as A, B, or C. Fisher’s exact test with a 5% two-sided significance level was performed to calculate the difference in response according to the presence or absence of PV spread. To minimise individual variance in PI absolute values, we calculated PI change ratios (PI at each time point—PI at T0/PI at T0) and temperature changes (temperature at each time point—temperature at T0) at T5, T15, and T30^14^. Pain severity and cold sensation over time were compared using the Wilcoxon rank sum test. The differences in PI change ratios and temperature changes over time were compared between groups using generalised estimating equations (GEE) analysis^14^. A *P* value of less than 0.05 was considered statistically significant.

### Ethics approval and consent to participate

This retrospective cohort study was approved by the Institutional Review Board (IRB) of Samsung Medical Center (approval No. 2019-10-046) and was registered with CRIS (Clinical Research Information Service of the Korea National Institute of Health, https://cris.nih.go.kr/cris/index.jsp, KCT0004405). The need for individual consent was waived by the IRB, as this was a retrospective study involving medical record review. All methods were performed in accordance with the relevant guidelines and regulations. All medical data were collected by a standardized protocol, and only analyzed after anonymisation.

## Results

Among the 88 patients assessed for eligibility, 32 were excluded owing to injections at L5. Two others were excluded owing to failed transforaminal injections consequent to epidural venography (n = 1) and distorted anatomy (n = 1). Finally, a total 54 patients were analysed. We defined responders as patients who showed a reduction of ≥ 50% on a NRS for pain at 30 min following block, and non-responders as those who showed a reduction of less than 50%. The demographic and clinical data are summarised in Table 1. The age, sex, duration of pain, lesion level, and lesion severity did not differ between the two groups (Table 1). The injection level, injection side, and epidural spread on lateral and AP views (*P* = 0.423, *P* = 0.073) also did not differ between the two groups (Table 2). No differences were observed between the two groups in the presence of PV spread (*P* = 0.082) and PV spread grade (A, B, C) (*P* = 0.312). Overall, PV spread was seen in 61.1% of patients, with 73.1% in responders and 50.0% in non-responders. In the PV spread analysis, 19 of 33 patients in the spread group were responders (57.6%) compared to 7 of 21 in the no spread group (33.0%). The post hoc power was 66.8% and the probability of type II error was 0.332. No differences were observed between the two groups in terms of PV spread status (*P* = 0.082), and grade (A, B, C) (*P* = 0.312). In both groups, the change in PI ratio differed significantly over time (from T5 to T30) (*P* = 0.002), but it was not different with respect to time and differing group (*P* = 0.821) (Table 3). The temperature change was not different over time (*P* = 0.139) or with respect to time and differing group (*P* = 0.328). The pain severity at T30 was different between the groups (*P* < 0.001) (Table 4). Cold sensation was not different between groups (Table 4). None of the patients showed any evidence of bleeding, dural puncture, or neurologic complications.

**Table 1:** Demographic and clinical characteristics of the patients.

	All patients (n = 54)	Responder (n = 26)	Non-responder (n = 28)	*P* value
Age, year	68.4 (9.4)	70.3 (6.8)	66.7 (11.1)	0.298
Sex, M/F	19/35	10/16	9/19	0.431
Diagnosis				0.024
Spinal stenosis	48 (88.9%)	26 (100.0%)	22 (78.6%)	
HNP	6 (11.1%)	0 (0.0%)	6 (21.4%)	
Duration of pain, months				0.857
< 3	6 (11.1%)	3 (11.5%)	3 (10.7%)	
3–12	19 (35.2%)	8 (30.8%)	11 (39.3%)	
> 12	29 (53.7%)	15 (57.7%)	14 (50.0%)	
Lesion level				0.879
L2–3	1 (1.9%)	1 (3.8%)	0 (0.0%)	
L3–4	16 (29.6%)	8 (30.8%)	8 (28.6%)	
L4–5	37 (68.5%)	17 (65.4%)	20 (71.4%)	
Lesion severity				0.586
Mild	0 (0.0%)	0 (0.0%)	0 (0.0%)	
Moderate	27 (50.0%)	12 (46.2%)	15 (53.6%)	
Severe	27 (50.0%)	14 (53.8%)	13 (46.4%)	

All data are presented as mean (SD) or numbers (percentages) of patients. HNP, herniated nucleus pulposus; Responder, patients who showed a reduction of ≥ 50% on the numeric rate scale for pain at 30 min after the block; Non-responder, patients who showed a reduction of less than 50%.

**Table 2:** Fluoroscopic data of the patients.

	All patients (n = 54)	Responder (n = 26)	Non-responder (n = 28)	*P* value
Injection level				0.208
L2	1 (1.9%)	1 (3.9%)	0 (0.0%)	
L3	9 (16.7%)	6 (23.1%)	3 (10.7%)	
L4	44 (81.5%)	19 (73.1%)	25 (89.3%)	
Injection side				
Left/right	25/29	11/15	14/14	0.571
Epidural spread				
Lateral view				0.423
Posterior	12 (22.2%)	7 (26.9%)	5 (17.9%)	
Anterior and posterior	42 (77.8%)	19 (73.1%)	23 (82.1%)	
Anteroposterior view				0.073
Extra-foraminal	17 (31.5%)	10 (38.5%)	7 (25.0%)	
Intra-foraminal	3 (5.6%)	3 (11.5%)	0 (0.0%)	
Extra-intra foraminal	34 (63.0%)	13 (50.0%)	21 (75.0%)	
Paravertebral spread				0.082
No spread	21 (38.9%)	7 (26.9%)	14 (50.0%)	
Spread	33 (61.1%)	19 (73.1%)	14 (50.0%)	
Pattern of spread				0.312
A	3 (9.1%)	2 (10.5%)	1 (7.1%)	
B	2 (6.1%)	1 (5.3%)	1 (7.1%)	
C	28 (84.8%)	16 (84.2%)	12 (85.7%)	

All data are presented as numbers (percentages) of patients. Presence of spread was defined as spread beyond the posterior margin of the vertebral body from the posterior epidural space observed in lateral view. Spread was graded A (beyond anterior 1/3), B (spread to middle 1/3), C (spread to posterior 1/3). Responder: patients who showed a reduction of ≥ 50% on the numeric rate scale for pain at 30 min after the block; Non-responder: patients who showed a reduction of less than 50%.

**Table 3:** Perfusion index change ratio and temperature change over time.

	Responder (n = 26)	Non-responder (n = 28)	*P* value time	*P* value time, group
PI change ratio			0.002	0.821
T5	1.8 (2.8)	1.1 (1.6)		
T15	2.5 (3.2)	1.0 (1.4)		
T30	2.7 (4.6)	0.7 (1.2)		
Temperature change			0.139	0.328
T5	− 0.1 (0.2)	− 0.0 (0.3)		
T15	− 0.1 (0.2)	− 0.0 (0.3)		
T30	− 0.2 (0.3)	− 0.1 (0.4)		

All data are presented as means (SD). PI: perfusion index, T0: before treatment; T5: 5 min following block, T15: 15 min following block, T30: 30 min following block, PI change ratio (PI at each time point—PI at T0/PI at T0), Temperature change (temperature at each time point—temperature at T0), Responder: patients who showed a reduction of ≥ 50% on the numeric rate scale for pain at 30 min after the block; Non-responder: patients who showed a reduction of less than 50%.

**Table 4:** Pain severity and cold sensation change over time.

	Responder (n = 26)	Non-responder (n = 28)	*P* value
**Pain severity, NRS**			
T0	7.0 (2.3)	6.7 (2.0)	0.584
T30	1.4 (1.7)	5.6 (1.6)	< 0.001
**Cold sensation, NRS**			
T0	2.3 (3.3)	2.0 (3.2)	0.655
T30	1.2 (2.2)	1.1 (2.3)	0.875

All data are presented as means (SD). NRS: numeric rate scale, T0: before treatment; T30: 30 min following block, Responder: patients who showed a reduction of ≥ 50% on the numeric rate scale for pain at 30 min after the block; Non-responder: patients who showed a reduction of less than 50%.

## Discussion

In the present study, we aimed to evaluate whether PV spread correlates with clinical efficacy in lumbar transforaminal block. Although the incidence of PV spread was higher in responders, there was no significant difference between the groups in terms of the evaluated parameters, including epidural spread pattern. We evaluated the clinical efficacy based on the PI change ratio and changes in temperature and cold sensation. In case of deactivation of the sympathetic nervous system, the PI may increase owing to decreased vasomotor tone and peripheral vasodilation^14,16^. We found that PV spread of the injectate did not correlate with the clinical efficacy in lumbar transforaminal block.

Lumbar radicular pain is caused by irritation or compression of the affected nerve root^17^. It is caused by narrowed neural foramina consequent to disc herniation, spinal stenosis, and degenerative spinal changes, resulting in a direct mass effect on the nerve root, as well as by inflammatory reactions^18^. The transforaminal approach provides more direct access into the neurotransforaminal space, and anterior epidural injection has been associated with superior analgesic outcomes for lumbosacral radicular pain^19,20^. PV block was first introduced in 1905 by Sellheim, and has gained popularity for acute and chronic pain in the thoracolumbar region^5,21^. The PV space is bounded by the vertebral body medially and by the transverse process posteriorly. The thoracic PV space continues caudally to the retroperitoneal space, which contains the lumbar plexus^4,5^. PV blocks may produce simultaneous somatic and sympathetic blockade by forwarding flow of injectate to the sympathetic chain^22^. The distance between the tip of the transverse process and lateral border of the intervertebral foramen in the thoracic PV space is approximately 1.5–2 cm; however, this may decrease to 1 cm or less at the T11 and T12 levels^5^. However, corresponding distances have not been reported in the lumbar area. We suspect that because of the proximity of the transverse processes and foramina, transforaminal injection may provide an opportunity for injectate spread to the PV space; however, this is subject to variations in patient anatomy or status of surrounding tissues. We speculate that, in our cohort, narrowed and/or distorted foraminal structures and consequent increases in intra-foraminal pressure may have affected injectate spread to the extra-foraminal space; however, as there was no difference in lesion severity between the groups, the incidence of PV spread was similar. Radicular pain may have several aetiologies, such as neural dysfunction, vascular compromise, inflammation, and biochemical influences, and it does not merely arise from neural compression due to foraminal narrowing^23,24^. Since the injectate may mostly flow around the dorsal root ganglion via the foramen, PV spread is unlikely to impact clinical efficacy. Morishita and colleagues reported that bony foraminal stenosis on imaging does not reflect the severity of clinical symptoms^25^.

This study had several limitations. First, we did not compare the volumes of injectate; therefore, the effect of volume on injectate spared could not be excluded. For lumbar transforaminal block, the reported injectate volume ranges from 0.2 to 9 ml^26,27^; yet we used 4–5 ml in our study. Second, we only planned approaches through the sub-pedicle area; therefore, we could not evaluate the extent of PV spread via other approaches, including retro-neural or retro-discal routes. Third, the lumbar sympathetic ganglia vary in number, size, and location, and presumably aggregate above L4^10^. In our study, the injection site was mostly at L4; therefore, the sympatholytic response may not have been expressed sufficiently. Fourth, our sample size was small, where the power was 66.8% and a risk of type II error was present. Further studies with larger sample sizes, improved power, and lower risk of type II errors are required to confirm our findings. Fifth, the follow-up period of 30 min was considerably inadequate for evaluating block efficacy. Sixth, we evaluated PV spread with 1–2 ml of contrast medium, and then by subsequent injection of 3 ml of local anesthetics and contrast medium. The two subsequent injections and differing viscosities between the contrast medium and other injectates may bias the evaluation of injectate spread. Finally, all patients had either received various analgesics, such as acetaminophen, ibuprofen, paracetamol, NSAIDs, opioids, and anticonvulsant^28^, or had received other interdisciplinary management protocols, which may have affected the severity of the pain after the block.

In our cohort, PV injectate spread did not correlate with successful pain relief following lumbar transforaminal block. Future prospective randomized studies are needed to determine whether larger volumes, different approach techniques, or injection at upper lumbar levels may affect PV spread during transforaminal block.

## Data Availability

The datasets generated during and/or analysed during the current study are available from the corresponding author on reasonable request.
